# A Panel of Serum MiRNA Biomarkers for the Diagnosis of Severe to Mild Traumatic Brain Injury in Humans

**DOI:** 10.1038/srep28148

**Published:** 2016-06-24

**Authors:** Manish Bhomia, Nagaraja S. Balakathiresan, Kevin K. Wang, Linda Papa, Radha K. Maheshwari

**Affiliations:** 1 Department of Pathology, Uniformed Services University of the Health Sciences, Bethesda, MD, 20814, USA; 2 Program for Neurotrauma, Neuroproteomics & Biomarker Research, Department of Psychiatry, McKnight Brain Institute, University of Florida, Gainesville, FL 32611, USA; 3 Department of Emergency Medicine, Orlando Regional Medical Center, Orlando, Florida, 32806, USA.

## Abstract

MicroRNAs (MiRNAs) are small endogenous RNA molecules and have emerged as novel serum diagnostic biomarkers for several diseases due to their stability and detection at minute quantities. In this study, we have identified a serum miRNA signature in human serum samples of mild to severe TBI, which can be used for diagnosis of mild and moderate TBI (MMTBI). Human serum samples of MMTBI, severe TBI (STBI), orthopedic injury and healthy controls were used and miRNA profiling was done using taqman real time PCR. The real time PCR data for the MMTBI, STBI and orthopedic injury was normalized to the control samples which showed upregulation of 39, 37 and 33 miRNAs in MMTBI, STBI and orthopedic injury groups respectively. TBI groups were compared to orthopedic injury group and an up-regulation of 18 and 20 miRNAs in MMTBI and STBI groups was observed. Among these, a signature of 10 miRNAs was found to be present in both MMTBI and STBI groups. These 10 miRNAs were validated in cerebrospinal fluid (CSF) from STBI and four miRNAs were found to be upregulated in CSF. In conclusion, we identified a subset of 10 unique miRNAs which can be used for diagnosis of MMTBI and STBI.

Traumatic brain injury (TBI) is a problem of epidemic magnitude involving both civilian, military service members and professional athletes. In the United States, more than 1.3 million emergency department visits account for TBI and is a cause of almost a third of all injury related deaths^1^. The economic burden of TBI in the United States is estimated to be $76.5 billion annually, in total lifetime direct medical costs and productivity losses^2^.

Mild TBI (MTBI) also called concussion accounts for more than 77% of the total reported TBI cases in the United States^3^. Among concussions, it is estimated that around 40% of these injuries are often ignored and do not seek medical attention. In addition, MTBI is also a major cause of morbidity in the veterans returning from the recent wars. It is reported that more than 20% of the veterans returning from the military engagements in Iraq and Afghanistan have experienced at least one episode of MTBI^4^. Most of the symptoms associated with MTBI resolve within days or weeks of injury with substantial recovery in most cases. However, approximately, 10–20% of MTBI patients complain of prolonged problems and some experience symptoms lasting more than a year. MTBI can induce neurological, cognitive and behavioral changes in an individual. The long term clinical symptoms may include headaches, sleep disturbance, impaired memory, anxiety and depression^5^. The accelerating and decelerating forces during the impact to the head results in the injury to the white matter causing diffuse axonal injury. Axonal injury may peak at 24 hr post injury and can progress up to a year post injury. It is believed that this continuous progression may be a causative factor for the poor outcome post MTBI^6^. MTBI usually is a challenge for the clinicians to diagnose because of the lack of apparent signs of a brain injury. MTBI is currently assessed using the Glasgow comma scale (GCS) which measure a score by assessing the eye movement, verbal, and motor response of the patient. GCS score and loss or alternations of consciousness are used to determine the severity of the injury^5^. The GCS score can be of limited use in MTBI diagnosis due to the presence of polytrauma, alcohol abuse, use of sedatives and psychological stress^7^. Computed tomography (CT) and magnetic resonance imaging (MRI) are used to detect the extent of brain injury, however in case of a concussion, CT and MRIs often fail to detect injury lesion due to limited sensitivity and absence of micro-bleeds. With new technological advancements, MRIs have become more sensitive than CT but due to their limited availability and high cost of the scan, the utilization of this technique is difficult for the acute stage diagnosis for both military and civilians^8^.

Biomarkers offer many advantages for MTBI diagnosis since they can be measured from the biofluids such as blood, urine and saliva and can be easily quantitated using standard biochemical and molecular methods. Several protein markers in serum and cerebrospinal fluid (CSF) such as S-100 calcium binding protein (S-100β), glial fibrillary acidic protein (GFAP) and Ubiquitin C-Terminal Hydrolase-L1 (UCH-L1) have been extensively studied for their utility as biomarkers for mild to severe TBI (STBI)^9^,^10^,^11^,^12^,^13^. However, most of the currently studied protein biomarkers have relatively low sensitivity for MTBI in individuals without detectable intracranial lesions. Combination of more than one protein biomarkers for MTBI diagnosis has been recently shown to have better diagnostic accuracy in comparison to single markers^14^. Despite extensive studies most of the protein markers are in preclinical or clinical testing and are not currently approved for clinical use.

MicroRNAs (miRNA) are small (19–28 nt) endogenous RNA molecules that regulate protein synthesis at the post transcriptional level^15^. MiRNAs can be detected in serum and can be an indicator of disease pathology in the cell of origin, including neuronal cells. This property of reflecting a diseased condition has gained attention for miRNAs as biomarkers of central nervous system (CNS) pathology^16^. Serum miRNAs are relatively stable at variable pH conditions, resistant to repeated freeze thaw and enzymatic degradation, which make them a suitable biomarker candidate for MTBI^17^.

MiRNAs have been reported as specific and sensitive biomarkers of many CNS diseases^18^. Utility of miRNAs as diagnostic markers of MTBI has been previously explored by our laboratory and others. We reported that expression of miRNA let-7i was upregulated both in serum and CSF after exposure to mild to moderate blast overpressure wave in a rodent model^19^. The serum expression of miRNAs in response to a concussive mild injury in a closed head injury model was also reported. A signature of 13 miRNAs was found to be modulated in the serum immediately after injury^20^. MiRNA modulation was also analyzed in a rodent model of traumatic stress. A signature of 9 miRNAs was identified which was upregulated in serum and amygdala of the animals 2 weeks after the exposure to traumatic stress. Interestingly, the 9 miRNAs from PTSD study and 13 miRNAs from TBI study were unique suggesting miRNA expression in serum may be a specific indicator of the altered physical state of the brain^21^. Redell and colleagues also studied the miRNA expression in human serum sample of TBI. Their results identified miR-16, miR-92a, and miR-765 as potential biomarkers of STBI with good diagnostic accuracy, however, the diagnostic accuracy of these markers for MTBI was limited^22^.

In this study, we have analyzed human serum samples of mild to moderate TBI (MMTBI) and STBI to discover miRNA candidates which can be used to diagnose milder injuries. The acute phase of TBI is the most critical time frame for diagnosis and supportive care; therefore, we have analyzed clinical serum samples from MMTBI and STBI subjects during the acute phase of the injury. The candidate biomarkers from the serum studies were validated in the CSF. Our study identified a unique miRNA signature which was present in all the serum samples irrespective of the grade of the injury. Further, analysis of the modulated miRNAs identified that these markers may play a very important role in neurological process in the brain.

## Materials and Methods

### Ethics Statement

Human samples were collected under approved institutional review board protocols at participating institutions including the Institutional Review Boards of Baylor University and University of Florida. All human subjects or their legal authorized representatives signed a consent form prior to enrollment. MicroRNA profiling experiments from human serum and CSF were performed at Uniformed Services University of the Health Sciences (USUHS) in accordance with the regulations and proper approval from the USUHS Institutional Review Board.

### Human Clinical Samples

Human STBI (GCS 3–8) serum (n = 8) and CSF (n = 8) samples were taken from an archived set of a completed STBI study (Baylor College of Medicine, NIH R01-NS052831-01)^11^. Serum samples were collected within 48 hr from injury with a mean of 33.8 hr post injury (range 12–48 hr) and CSF samples (n = 8) were collected within 48 hr of injury (mean 26.3 hr). Human serum samples of MMTBI (n = 8) (GCS 9–15) and orthopedic injury patients (n = 7) who did not have head injury were collected within 24 hr of injury (mean 3.1 hr post-injury with a range of 0.5–12 hr)^23^. Normal healthy control serum (n = 8) and normal control CSF (n = 6) samples were obtained from Bioreclamation Inc. for comparison.

### RNA Isolation

RNA isolation was performed using miRNeasy Serum/Plasma Kit (Qiagen Inc) according to the manufacturer’s instruction. Briefly, frozen serum samples were thawed on ice and 200 μl of serum was added to QIAzol lysis reagent (1 ml) and vortexed. Chloroform (200 μl) was added and samples were incubated at room temperature for 5 min. Tubes were centrifuged for 15 min at 12,000 × g at 4 °C. The aqueous phase obtained after centrifugation was mixed with 1.5 volumes of 100% ethanol. This mixture (700 μl) was loaded into an RNeasy minElute spin column in a 2 ml collection tube and followed by centrifugation. The column was sequentially washed with Buffer RWT (700 μl), Buffer RPE (500 μl), 80% ethanol (500 μl) and then RNA was eluted with RNase-free water (14 μl).

### RNA quality control

All total RNA samples were analyzed with the Agilent Small RNA kit (Agilent Technologies Inc, Santa Clara, CA, USA) to measure the small RNA/miRNA concentration. 1.5 μl aliquots of total RNA samples were heat denatured for 2 min at 70 °C. Agilent Small RNA kit was used for determining the small RNA concentration, miRNA concentration and also the small RNA/miRNA ratio in the given RNA samples using a specific small RNA chip. The small RNA chip was prepared according to the manufacturer’s protocol. Briefly, the gel-dye mix was prepared by mixing 40 μl of the filtered gel matrix with 2 μl of the dye concentrate and spinning the mixture at 13,000 g for 10 min at room temperature. The small RNA chip was loaded with the gel-dye mixture using the standard chip priming technique followed by addition of 9 μl of Small RNA conditioning solution to the chip. 5 μl of the small RNA marker was added to each sample well. 1 μl of the RNA ladder was loaded into the assigned ladder well. 11 denatured RNA samples (1 μl each) in the concentration range from 1 to 100 ng/μl was loaded into the sample wells of the chip. The chip was vortexed, and run on the Agilent 2100 bioanalyzer.

### Reverse Transcription and pre-amplification

Reverse transcription (RT) was performed with TaqMan miRNA RT Kit (Life Technologies, Carlsbad, CA, USA) as described with slight modifications^19^. MiRNA quantity was measured from the total RNA of bioanalyzer data and used as template RNA (30 ng-serum miRNA) for RT reactions. Briefly, RT reaction mixture contained 0.8 μl megaplex RT primers human pool A/B (v3.0), 0.2 μl 100 mM dNTPs (with dTTP), 1.5 μl multiscribe reverse transcriptase (50 U/μl), 0.8 μl 10X RT Buffer, 0.9 μl MgCl_2_ (25 mM), 0.1 μl RNAse inhibitor (20 U/μl), RNA template and nuclease free water to a final volume of 7.5 μl. RT reaction was carried out on Veriti 96-Well Thermal Cycler (Life Technologies, Carlsbad, CA, USA) according to manufacturer’s recommended thermal cycling conditions.

Pre-amplification of the cDNA product after RT was done using 12.5 μl TaqMan preAmp Master Mix, 2.5 μl megaplex preamp primers human pool A/B (v3.0), 5 μl of nuclease-free water and 5 μl of RT product to make up a final volume of 25 μl of final reaction mixture. Pre-amplification reaction was carried out on a Veriti 96-Well Thermal Cycler (Applied Biosystems/Life Technologies, Carlsbad, CA, USA) according to the following thermal cycling conditions: 95 °C for 10 min, 55 °C for 2 min, 72 °C for 2 min followed by 14 cycles of 95 °C for 15 sec and 60 °C for 4 min, followed by a hold at 99.9 °C for 10 min and a final hold of 4 °C.

### Taqman Low Density Array -Real Time PCR

To prepare the real time PCR reaction mix, 9 μl of undiluted pre-amplification product was added to 450 μl of 2X TaqMan Universal PCR Master mix, no AmpErase UNG (Applied Biosystems Inc.) and nuclease free water was added to a final volume of 900 μl. 100 μl PCR reaction mix was loaded onto each row of the 384-well TaqMan Low Density Human MicroRNA array cards (TLDA). The PCR reaction was carried out at default thermal-cycling conditions in AB7900 Real Time HT machine (Applied Biosystem). Profiling of miRNAs was carried out using TaqMan^®^ human microRNAs Array Set v3.0 (Life Technologies, Inc.).

### Specific MiRNA assays for serum and CSF samples

TaqMan MicroRNA assay (Life Technologies, Inc.) was used for specific miRNA assays. 50 ng of total RNA of serum (MMTBI and STBI) or 20 ng for CSF (severe) samples, was used to perform RT using miRNA specific primers for five candidate (miR-195, miR-328, miR-362-5p, miR-486 and miR505*) and for endogenous control (miR-202) as per manufacture’s protocol (Life Technologies Inc.). MiR-202, which gave most stabilized expression both in control and TBI samples, was used as an endogenous control for the validation of all selected candidate miRNAs. For CSF samples, a pre-amplification step was performed. Thereafter, real-time qPCR was performed using a TaqMan MicroRNA assay to quantitate individual miRNAs. For CSF samples, a pre-amplification step was performed with the RT product due to low miRNAs concentration. Briefly, 5 μl of RT product was used along with 10 μl of TaqMan preamp mastermix along with 5 μl 0.2X TaqMan small RNA assay (20x). The thermal cycling conditions: 95 °C for 10 min, 55 °C for 2 min, 72 °C for 2 min followed by 14 cycles of 95 °C for 15 sec and 60 °C for 4 min, followed by a hold at 99.9 °C for 10 min and a final hold of 4 °C. TaqMan miRNA assays were carried out to validate the changes in level of expression for the TBI candidate miRNAs. TaqMan miRNA assays were carried out in triplicates. Each miRNA was calibrated to a selected endogenous control miR-202 to get a delta Ct (ΔCt) value for each miRNA (miRNA Ct value–miR-202 Ct value). The fold changes were then calculated using the comparative Ct method (2^−ΔΔCt^).

### Statistical analysis

MiRNA expression profiles for raw cycle threshold (C_t_) values were analyzed using real-time Statminer software (Integromics, Inc) to identify significantly altered miRNAs. For relative quantification of miRNAs between control and TBI samples, the following steps were performed in the Statminer software suite: quality control of biological replicates, filtering of miRNAs expression having Ct values below 35 cycles and the detection of expression in all biological replicates of calibrator and target. Statistically significant miRNAs were selected based on the following stringent parameters such as Benjamin-Hochberg false discovery rate (FDR) corrections conservatively select data with adjusted p-values and p-value lower than both 0.01 and 0.05. Functional pathway analysis of altered miRNAs and their association with TBI related gene targets were performed using Ingenuity Pathway Analysis (IPA) program (Ingenuity Systems Inc., Redwood City, CA). For clinical correlation analysis, data comparing changes in miRNA expression are described as means with standard error of the mean. In order to analyze the differences between group means we used analysis of variance (ANOVA) after the assessing for distribution and variance. Multiple comparisons were performed using Games-Howell test. Significance was set at 0.05.

## Results

All TBI patients were older than 18 years old and had a non-penetrating brain injury with a GCS score of 3 to 15. Those with STBI had a GCS <= 8 and required the placement of an intraventricular catheter (IVC). Those with mild to moderate TBI (MMTBI) had a GCS 9–15 and did not have an intraventricular catheter (IVC) placed. The mean age of severe TBI patients was 39 (SD17) years and 100% were male. The mean age of MMTBI patients was 36 (SD16) years, 88% were male. Seventy five percent (75%) of MMTBI patients had a GCS 14-15.

### Identification of miRNA biomarkers

We performed real time PCR for a set of 792 human miRNAs for serum samples of TBI (n = 8), STBI (n− = 8), orthopedic injury (n = 7) and healthy controls (n = 8). PCR amplification of the miRNAs in human serum detected more than 140 miRNAs in the control serum samples, which is consistent with the previously reported findings^24^. For relative quantitation of miRNAs in serum samples, a stable endogenous control is a major limitation. We analyzed the expression levels of the previously reported endogenous controls such as U6 snRNA, RNU 44, RNU 48 and miR-16, using genorm algorithm. This analysis showed a high variability of the expression levels of these molecules between the study groups. Therefore, to analyze the real time PCR miRNA data, a global normalization algorithm was used which calculates a reference endogenous control based on the overall amplification of the miRNAs in the same plate. This method has been used by others as a way of normalization for multiplexing assays in serum samples^20^.

**Figure 1:**
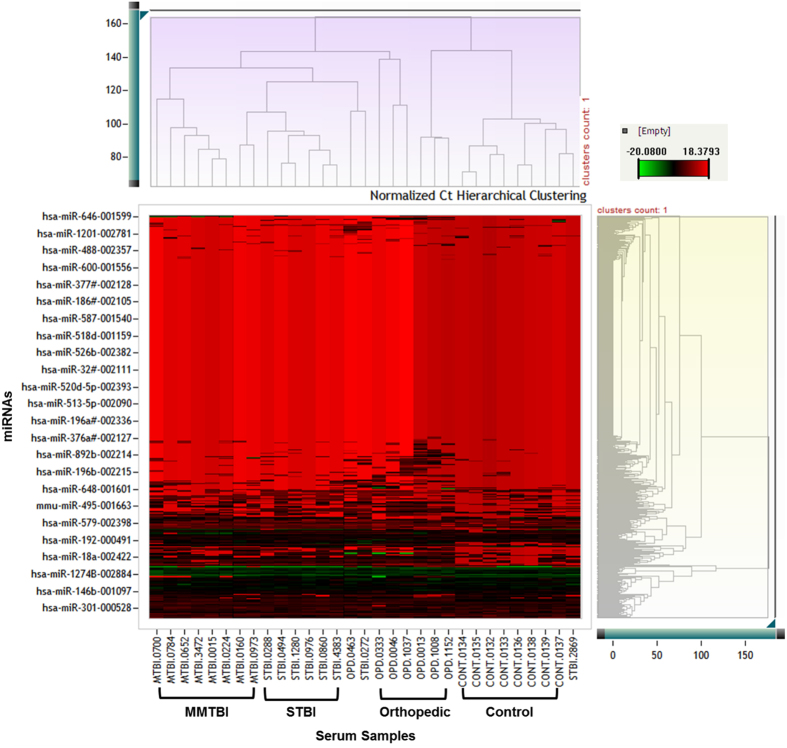
Hierarchical clustering of the total miRNA profile of all the samples using their delta Ct values to understand the pattern of expression in different experimental groups. The dendrogram was constructed by hierarchical clustering of normalized gene expression heat map data (DCt) value(s) of 758 detectors (754 miRNAs and 4 endogenous controls) and 31 serum samples using complete linkage method and Euclidean distance. The red and green colors in the heat map representing up and down -regulated gene expression levels, respectively. Control and TBI groups show distinct changes.

We performed hierarchical clustering with the normalized delta Ct values to understand pattern of expression between the experimental groups. Clustering analysis showed a segregated data under four differentially expressing groups which belonged to control, orthopedic injury and the TBI groups suggesting overall difference in miRNA expression between these experimental groups (Fig. 1). After the normalization, the fold change for the serum miRNAs in MMTBI, STBI and orthopedic injury groups was calculated using healthy control subjects as baseline. MiRNAs with more than 2 fold upregulation and adjusted p value <0.05 were selected for further analysis. From this analysis, it was found that in serum samples of MMTBI and STBI, 39 and 37 miRNAs were significantly upregulated respectively whereas 33 miRNAs were found to be modulated in orthopedic injury group (Supplementary Table 1–3). MiRNAs with reduced expression in the TBI groups may not be suitable as biomarkers for detection of MMTBI, therefore these miRNAs were excluded from further analysis.

**Figure 2:**
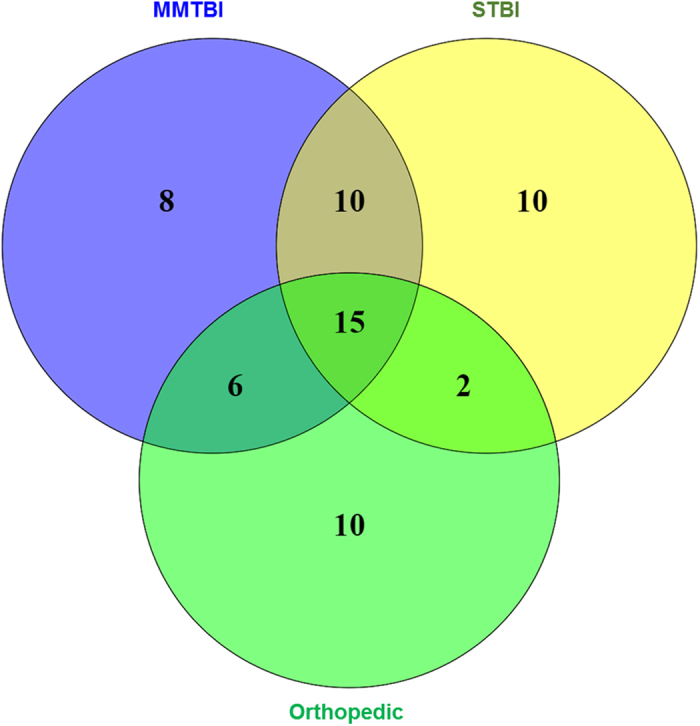
Venn diagram showing significant expression of miRNAs in MTBI, STBI and orthopedic injury control groups in comparison to control samples. MiRNA expression was normalized using global normalization algorithm. Each of the injury group was normalized with the control samples to identify significantly modulated miRNAs in injury groups.

To identify miRNAs specific for the TBI groups, a comparison between MMTBI and STBI vs orthopedic injury was performed. From this comparison, 18 miRNAs in MMTBI group and 20 miRNAs in STBI groups were identified (Tables 1, 2). A comparison was performed between the miRNAs upregulated in both MMTBI and STBI group which identified a signature of 10 miRNAs viz miR-151-5p, miR-195, miR-20a, miR-328, miR-362-3p, miR-30d, miR-451, miR-486, miR-505* and miR-92a, with increased expression in both MMTBI and STBI groups (Fig. 2, Common miRNAs in MMTBI and STBI are highlighted in bold in Tables 1 and 2). Among these miR-92a has been previously reported as a biomarker for STBI but it was not found to be sensitive biomarker for MMTBI^22^.

### Validation of candidate miRNAs

**Figure 3:**
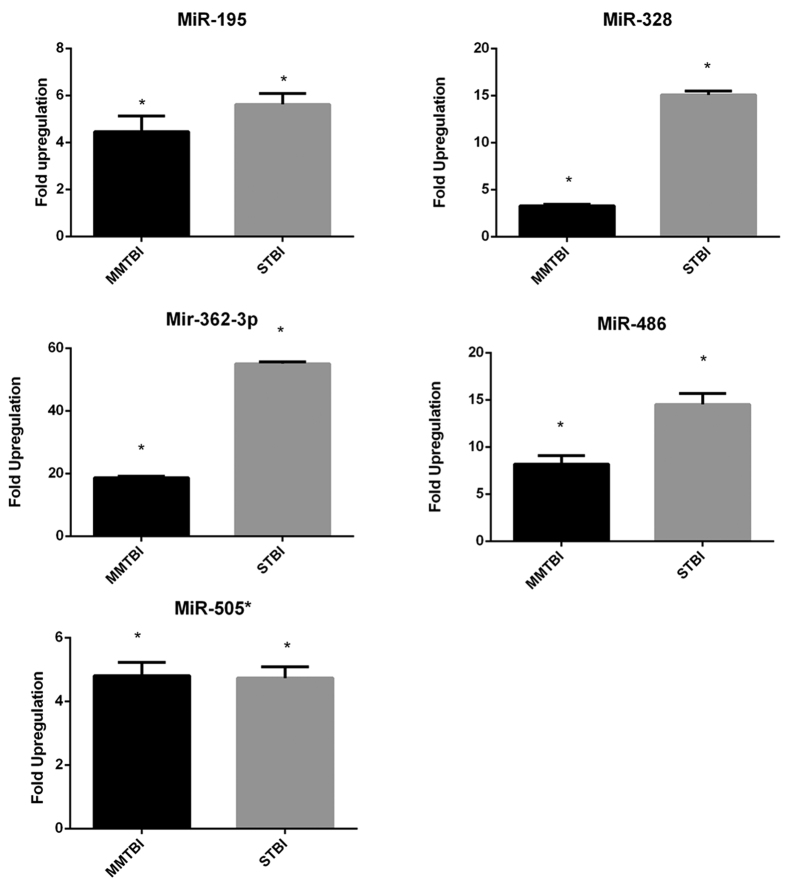
MiRNA specific validation assays in serum samples of MMTBI and STBI. To validate the data from the multiplex screening, individual miRNA PCR assays were performed. Normalization was done with mir-202 which showed the least standard deviation and was selected as a normalizing control. Values are expressed as fold change +SEM over control in linear scale. The statistical significance was calculated using paired student t test (p < 0.05) using the individual delta Ct value for the samples of control and the injury groups.

To validate the findings of miRNA screening using TLDA platform specific miRNA PCR assays were performed for selected miRNAs. The validation step is performed because miRNAs screening using TLDA platform has a possibility of introducing a bias in expression of miRNAs because of a pre-amplification step of the cDNA before the RT-PCR^19^. We randomly selected five miRNAs miR-195, miR-328, miR-362-3p, miR-486 and miR-505* and performed specific miRNA assays to validate their expression. In order to perform the specific miRNA PCR assays, a stable endogenous control is required. During the TLDA screening, we used global normalization algorithm for normalization which we used in conjunction with equal starting volume of the serum. For specific assays, a stable endogenous control was identified by selecting the miRNA with the least standard deviation in the delta Ct values obtained after global normalization. By this method, we identified that miR-202 was stably expressed among all the injury groups as well as in the control group and therefore it was selected as endogenous control for all the subsequent experiments. To validate the miRNA expression data we selected the 10 common miRNAs which were upregulated in both MMTBI and STBI groups. For validation experiments, RNA was isolated from the serum samples and assays were performed without pre-amplification of cDNA. The validation assays showed significant upregulation of the miRNAs in both MMTBI and STBI groups when compared to the control samples consistent with our results of miRNA profiling (Fig. 3). Our results demonstrated that all the selected miRNAs were significantly upregulated in after TBI; however, their fold upregulation values were not same as in the initial expression profiling. This difference can be possibly attributed to the use of a pre-amplification step in the multiplex profiling.

### Presence of miRNAs in cerebrospinal fluid

A biomarker of mild or severe traumatic brain injury should reflect the injury to the brain cells such as neurons or glial cells. An injury to these cells will lead to secretion of molecules such as proteins and miRNAs either native form or encapsulated in small vesicles called exosomes into the blood and CSF. To validate the presence of miRNAs observed in serum studies, a complete miRNA profiling was performed using CSF samples from STBI patients (n = 8) and control CSF samples (n = 6). From our initial CSF sample profiling, it was observed that the average number of total miRNAs which were detected per sample was approximately 75 with very high Ct values. The reason for the poor performance of global profiling was possibly because of low miRNA content of CSF and limiting sample volume in our studies. Due to these limitations, we only performed specific miRNA assays for the ten candidate miRNAs identified in serum as biomarker candidates in both MMTBI and STBI groups.

**Figure 4:**
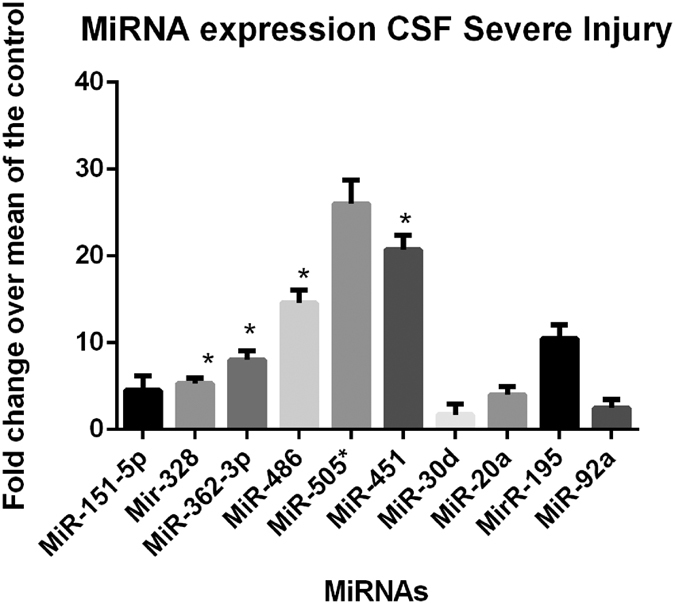
MiRNA specific validation assays in CSF samples of STBI. Specific miRNA assays were performed for the five candidate miRNAs. Normalization was done with miR-202 which showed the least standard deviation and was selected as a normalizing control. Values are expressed as fold change +SEM over control in linear scale. The statistical significance was calculated using paired student t test (p < 0.05) using the individual delta Ct value for the samples of control and the injury groups.

The conventional miRNA assay methodology was modified and an additional pre-amplification step was added in the analysis. This pre-amplification does not introduce additional bias since only one primer is used for pre-amplification reaction. The real time data for miR-151-5p, miR-195, miR-20a, miR-30d, miR-328, miR-362-3p, miR-451, miR-486, miR-505* and miR-92a was normalized using miR-202. MiR-202 was found extremely stable in the CSF samples with a mean Ct value of 26.2 and 25.8 in injury and control samples respectively. Normalization with miR-202 showed a significant upregulation of miR-328, miR-362-3p, miR-451 and miR-486 (Fig. 4). For miR-505* and miR-195, although the mean fold upregulation was more than 10 fold, however it was only observed in 50–60% of the samples whereas in the remaining samples it was not detected, hence these failed the statistical test. Similar observation was also found for miR-20a. An increase in miR-151-5p was observed, but it was not significant due to sample outliers. No significant upregulation in the level of miR-30d was observed between control and injury groups.

### MiRNA biomarker correlation with CT diagnosis

**Figure 5:**
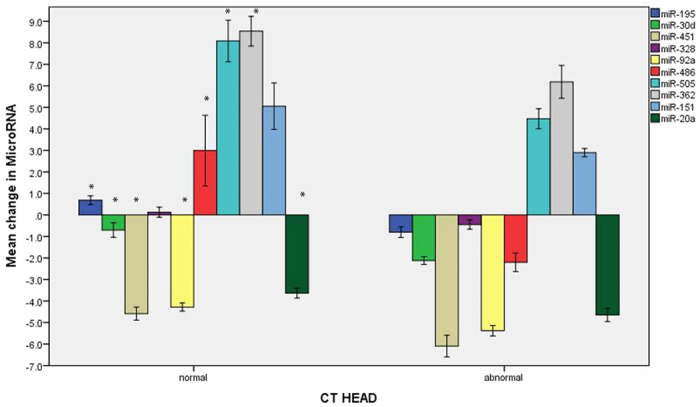
Levels of MicroRNA Biomarkers In Those With Head Ct Lesions Versus No Head Ct Lesions: Comparison of levels of miRNA in two groups of human subjects. Group 1 is comprised of subjects (TBI and controls) without any lesions on head CT (n = 19). Group 2 is TBI subjects with lesions on head CT (n = 12). The assumption was made that all controls (normal and trauma) had negative CT scans. There were significant differences between the two groups for all but two of the selected miRNA (see asterisks): miR-195 (p < 0.001); miR-30d (p < 0.001); miR-451 (p < 0.011); miR-328 (p = 0.101); miR-92a (p < 0.001); miR-486 (p = 0.006); miR-505 (p = 0.008); and miR-362 (p = 0.035); miR-151 (p = 0.065); and miR-20a (p = 0.012).

In order to identify a correlation of miRNAs with the CT lesions we analyzed the miRNA data with the delta Ct data from the real time PCR data of the TBI and trauma control groups. The comparison between these groups was performed using the delta Ct values because of the absence of absolute fold change. A comparison of level of miRNA was performed in 2 groups of human subjects comprised of 1) subjects (TBI and all controls) without any lesions on head CT (n = 19); and 2) TBI subjects with lesions on head CT (n = 12). The assumption was made that all normal and trauma controls had negative CT scans. There were significant differences between the two groups for all but two of the selected miRNA: miR-195 (p < 0.001); miR-30d (p < 0.001); miR-451 (p < 0.011); miR-328 (p < 0.101); miR-92a (p < 0.001); miR-486 (p < 0.006); miR-505 (p < 0.008); and miR-362 (p < 0.035); miR-151 (p < 0.065); and miR-20a (p < 0.012) (Fig. 5).

### Diagnostic accuracy of the miRNAs

**Figure 6:**
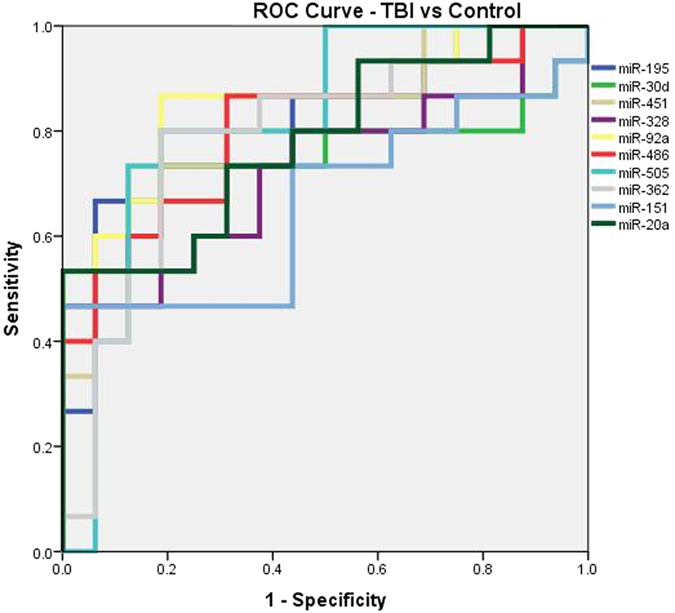
The diagnostic accuracy was assessed using the ROC Curve to determine the area under the curve for distinguishing TBI from controls. The AUC’s were: miR-195 (0.81), miR-30d (0.75), miR-451 (0.82), miR-328 (0.73), miR-92a (0.86), miR-486 (0.81), miR-505 (0.82), miR-362 (0.79), miR-151 (0.66), miR-20a (0.78).

Receiver operator characteristic (ROC) curve was generated to calculate the area under the curve (AUC) to identify the accuracy of the miRNAs in diagnosing TBI. The analysis identified the AUC values as miR-195 (0.81, p value < 0.003), miR-30d (0.75, p value < 0.016), miR-451 (0.82, p value < 0.002), miR-328 (0.73, p value < 0.030), miR-92a (0.86, p value < 0.001), miR-486 (0.81, p value < 0.003), miR-505 (0.82, p value < 0.002), miR-362 (0.79, p value < 0.006), miR-151 (0.66, p value < 0.123), miR-20a (0.78, 0.007). All miRNAs except for miR-151 showed good diagnostic accuracy (Fig. 6).

### Computational analysis of mRNA targets of miRNAs

**Figure 7:**
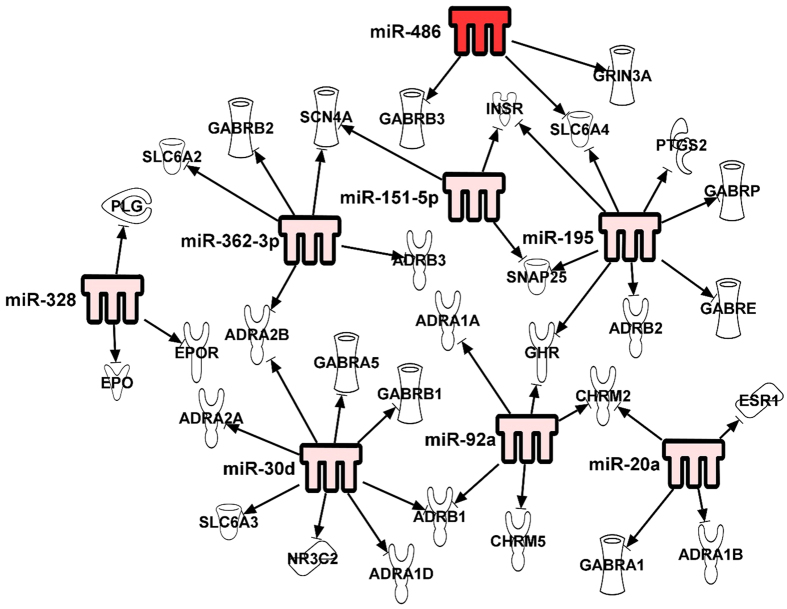
Ingenuity pathway analysis program identified direct targets for TBI miRNA candidates. The red color indicates the miRNAs upregulated expression.

MiRNAs target mature mRNAs and regulate the protein expression. To understand a possible regulatory role of the TBI biomarker candidates identified from the initial expression profiling, miRNAs were analyzed for their association with TBI related gene targets using IPA. In IPA, there are currently 87 target molecules whose association has been linked with miRNA regulation in TBI. Therefore, we used the eighty seven TBI related molecules which are available in the disease and function category and identified any direct relation of these targets with the 10 candidate miRNAs. The pathway explorer function of IPA was used to build putative pathways between TBI miRNA biomarker candidates and TBI related molecules. This analysis identified 30 genes as direct targets for the 8 miRNA candidate miR-151-5p, miR-195, miR-328-3p, miR-362-3p, miR-30d, miR-20a, miR-486 and miR-92a. MiR-505* and miR-451 were not predicted to target any of the target molecules for TBI in IPA. These genes were further analyzed by overlying them in the canonical pathway category. This analysis identified that most of the molecules predicted to be targeted by the miRNAs are involved in major TBI related canonical pathways such as erythropoietin signaling, G protein coupled receptor signaling, GABA receptor signaling, and neuropathic pain signaling in dorsal horn neurons. Specifically, miR-328 was predicted to regulate erythropoietin and erythropoietin receptor which are important mediators of erythropoietin signaling. MiR-486, miR-27a and miR-195 targeted molecules involved in glutamate receptor signaling and GABA receptor signaling. MiR-151-5p and miR-362-3p target molecule SCN4A which is shown to be responsible for generation and propagation of neurons. MiR-30d was also predicted to target adrenoceptors and GABA receptor signaling. Overall, it was found that all the most of the miRNAs target important neurological pathways (Fig. 7).

## Discussion

Several past and ongoing research efforts are directed towards identifying a clinically reliable diagnostic biomarker for MMTBI. Much of the efforts have been directed towards identifying blood based protein biomarkers of TBI^25^. Many of the protein markers which have shown promise in cases of moderate to STBI are still not approved for clinical use. One of the possible reasons for lack of a sensitive and specific blood based protein biomarker for MMTBI is due to limitations of proteins in traversing through the blood brain barrier after injury due to their large molecular weight. Other reasons may include susceptibility to degradation by endogenous proteases. Moreover, low proteins concentrations are also sensitive to laboratory handling conditions and which may further make it difficult to develop a sensitive blood based protein biomarker for MMTBI.

MiRNAs are relatively abundant and stable in biofluids. Due to these properties miRNA has advantages over protein based markers and are now being studied as the next generation of biomarkers for many diseases and disorders such as cancer, cardiovascular disorders and neurodegenerative diseases^26^. Our laboratory has previously reported miRNA let-7i as a biomarker of blast induced mild -moderate TBI^19^. A signature of 13 miRNAs was identified in mouse serum as a biomarker of mild closed head injury^20^. These two studies identified unique miRNA signatures following two completely different injuries. Our recent study also found that miRNA modulation in a PTSD model was unique and had no resemblance to the TBI miRNA profile in serum^21^. In a previous report, two miRNAs, miR-16 and miR-92a were identified using a microarray based screening method in human plasma samples after STBI and these were reported as specific and sensitive markers of STBI. However, their diagnostic potential was found to be limited for MMTBI. Several other studies have also reported miRNA modulation in brain post TBI in different rodent models^27^,^28^.

In this study, our aim was to identify specific and sensitive miRNA based biomarkers for MMTBI using a more specific and sensitive real time PCR methodology. Serum samples from human subjects with STBI (CSF and serum), MMTBI (serum), orthopedic injury (serum) and no injury controls (CSF and serum) were used in this study. Inclusion of the orthopedic injury group was important because head trauma can also accompany other orthopedic injuries and an orthopedic injury by itself may lead to alteration of miRNAs. The serum samples were collected within 12 hr and 48 hr for MMTBI and STBI, respectively. The sample collection time is crucial for biomarker identification for MMTBI because the acute phase of the injury presents the best opportunity for identifying a blood based cerebral injury marker.

MiRNA expression profiling was performed using real time PCR based platform which is currently considered as the most sensitive platform for miRNA analysis. The initial analysis consisted of a clustering step to understand the difference in the total miRNA expression between the non-trauma controls and trauma group. Clustering analysis showed that miRNA expression patterns were similar within the TBI group where samples from MMTBI and STBI were clustered together whereas the control and orthopedic injury samples were segregated separately. This initial evidence showed that the overall miRNA expression profile can be an indicator of the brain injury. Real Time PCR based screening of miRNAs using TLDA platform identified 39 and 37 significant upregulated miRNAs in MMTBI and STBI serum samples, respectively. However, the miRNA profile of orthopedic injury identified 33 significantly modulated miRNAs which shared a considerable level of similarity with the MMTBI and STBI profiles. This observation is not surprising because often times a peripheral orthopedic injury is associated with a traumatic brain insult and vice versa. Therefore, we identified specific miRNAs for MMTBI and STBI by comparing the profile with the orthopedic injury. This comparison identified 18 and 20 miRNAs in MMTBI and STBI groups respectively.

Next we focused on identifying the common miRNAs between the MMTBI and STBI groups. The analysis identified 10 miRNAs (Tables 1 and 2) with significant elevated expression in both MMTBI and STBI. Common miRNAs between the injury groups were identified because a marker found in MMTBI should ideally be expressed at elevated levels in STBI. We performed validation of randomly selected 5 miRNAs candidates in the serum without performing the pre-amplification step. Expression levels of the 5 miRNAs after TBI was found to be upregulated in both MMTBI and STBI groups. The fold changes in the validation experiments did not exactly match with the TLDA profiling data however, this may be due to the use of pre-amplification step in the TLDA profiling. Moreover, the Ct values in control group for miR-362-3p were found to be very high (>35) suggesting their expression in control samples is negligible, however in injury groups their expression level considerably increased, which resulted in very high fold changes over the control. Nevertheless, the validation experiments confirmed the data from the multiplex array platform in both MMTBI and STBI groups.

Expression level of the miRNA candidates was also evaluated in the CSF samples obtained from STBI subjects within 48 hr of injury. CSF is considered to have all the secreted molecules and metabolites from the neurons. Therefore, we hypothesized that the CSF should have a higher concentration of brain injury related molecules. Validation in CSF samples showed that expression of miR-328, miR-362-3p miR-486 and miR-451 was significantly upregulated, however; no significant elevation in levels of miR-151-5p, miR-30d and miR-20a was detected. Among them, miR-151-5p was upregulated by more than 4 fold but due to sample outliers this was not significant. MiR-505* and miR-195 were upregulated however due to sample outliers in a small group it was not statistically significant. Overall, the validations experiments in CSF showed a similar trend of miRNA expression as seen in serum, but due to limited samples size statistical significance for some of the miRNAs was not achieved.

MiRNA data was also compared with the head CT data of the trauma subjects with lesions and without CT lesions. Other than miR-151-5p and miR-328, the remaining 8 miRNAs showed a direct and statistically significant correlation with the increased miRNA expression in case of subjects with CT lesions. This suggests that, with increasing grade of injury which is evident by the presence of lesions on CT, more miRNAs are secreted in the serum, which is an indirect measure of severity of the injury to the neural tissue. Further, to evaluate the diagnostic accuracy of these miRNAs, we performed ROC curve analysis and calculated the AUC values. Except of miR151-5p, other miRNAs performed well in the diagnostic accuracy with AUC values ranging from (0.73–0.86, 95% CI). Computational analysis was also performed understand the relationship of the 10 miRNA biomarkers with that of important regulatory molecules implicated in TBI. Our analysis using IPA showed that, 7 miRNAs are experimentally validated to target important TBI related proteins. Some of these proteins include erythropoietin, adrenoreceptors and GABA receptors which are known play an important role in TBI pathology. These observations suggest that miRNAs may be directly involved in TBI related pathology.

MiRNAs have been previously studied in rodent models and human TBI. MiR-16 and miR-92a were reported as biomarkers of STBI in humans^22^. In our study, miR-16 and miR-92a were significantly upregulated in both MMTBI and STBI. However, miR-16 was also found to be significantly upregulated in orthopedic injury group suggesting that miR-16 may not be only specific to TBI. Comparison of miRNAs modulated in this study with that of serum miRNA from blast induced MMTBI in rats show common miRNAs such as miR-20a, miR-362-3p, miR-195, miR-451 and miR-92a. MiR let-7i upregulation in serum after blast induced TBI was not observed in this study. MiR-362-3p identified in this study was likewise found to be modulated in a rodent model of closed head injury^20^. MiR-451 was reported to be upregulated in the cortex after a TBI in a mouse model^29^. Overall, the some miRNAs identified in this study correlate with the previously identified miRNAs in rodent model however many miRNAs were unique to this study and were not previously reported as TBI biomarker. Differences in the expression of serum miRNAs in rodent and human studies may be due to the heterogeneity of the TBI in humans in comparison to a controlled TBI such as blast injury in animal studies.

Moreover, miRNAs modulation in other neurological disorders that leads to neurodegeneration such as stroke can be compared with the TBI. Stroke leads to injury of the brain and additional studies can provide better understanding of miRNAs in neuronal injury. In fact, in one of the studies, miR-92a levels were increased in CSF and blood of the patients who suffered an aneurysmal subarachnoid hemorrhage^30^. Further studies in other neurodegenerative disorders can be performed to further evaluate the specificity of miRNAs for TBI.

In conclusion, we have identified a 10 miRNA signature panel in serum that corresponds to MMTBI. These miRNAs can be tested in larger human clinical study to further evaluate their sensitivity and specificity for clinical diagnosis of MMTBI. Varied injury samples consisting of military and civilian TBI cases are needed to test the sensitivity of these candidate miRNAs in the detection of TBI caused by blunt head trauma vs non- impact primary blast overpressure wave injuries. Studies are in progress to determine the potential of these miRNAs as specific biomarkers of MMTBI in larger sample size. To our knowledge, this is the first study to report these miRNAs as potential clinical biomarkers of human mild traumatic brain injury.

## Additional Information

**How to cite this article**: Bhomia, M. *et al*. A Panel of Serum MiRNA Biomarkers for the Diagnosis of Severe to Mild Traumatic Brain Injury in Humans. *Sci. Rep.* **6**, 28148; doi: 10.1038/srep28148 (2016).

## Supplementary Material

Supplementary Information

## Figures and Tables

**Table 1:** MiRNAs altered in serum samples of MMTBI.

S. No	miRNA	MIMAT ID	Mature sequence	Fold Upregulation
1	hsa-miR-1255B	MIMAT0005945	CGGAUGAGCAAAGAAAGUGGUU	19.19
2	**hsa-miR-151-5P**	MIMAT0004697	UCGAGGAGCUCACAGUCUAGU	45.52
3	hsa-miR-194	MIMAT0000460	UGUAACAGCAACUCCAUGUGGA	31.43
4	**hsa-miR-195**	MIMAT0000461	UAGCAGCACAGAAAUAUUGGC	2.46
5	hsa-miR-199a-3p	MIMAT0004563	ACAGUAGUCUGCACAUUGGUUA	3.02
6	**hsa-miR-20a**	MIMAT0000075	UAAAGUGCUUAUAGUGCAGGUAG	4.19
7	hsa-miR-27a	MIMAT0000084	UUCACAGUGGCUAAGUUCCGC	2.06
8	hsa-miR-27b	MIMAT0000419	UUCACAGUGGCUAAGUUCUGC	2.51
9	**hsa-miR-30d**	MIMAT0000245	UGUAAACAUCCCCGACUGGAAG	2.92
10	**hsa-miR-328**	MIMAT0000752	CUGGCCCUCUCUGCCCUUCCGU	2.56
11	**hsa-miR-362-3p**	MIMAT0004683	AACACACCUAUUCAAGGAUUCA	14.54
12	hsa-miR-381	MIMAT0000736	UAUACAAGGGCAAGCUCUCUGU	2255.75
13	**hsa-miR-486**	MIMAT0002177	UCCUGUACUGAGCUGCCCCGAG	523.46
14	hsa-miR-505*	MIMAT0004776	GGGAGCCAGGAAGUAUUGAUGU	33.39
15	hsa-miR-625*	MIMAT0004808	GACUAUAGAACUUUCCCCCUCA	40.51
16	hsa-miR-638	MIMAT0003308	AGGGAUCGCGGGCGGGUGGCGGCCU	46.48
17	**hsa-miR-92a**	MIMAT0000092	UAUUGCACUUGUCCCGGCCUGU	3.77
18	**mmu-miR-451**	MIMAT0001631	AAACCGUUACCAUUACUGAGUU	8.37

Data was normalized using global normalization and was compared with healthy controls and orthopedic injury samples. Data was adjusted for multiple comparisons using adjusted p value  ≤ 0.05 calculated using Benjamin Hochberg algorithm.

**Table 2:** MiRNAs altered in serum samples of STBI.

S. No	miRNA	MIMAT ID	Mature sequence	Fold Upregulation
1	hsa-miR-1291	MIMAT0005881	UGGCCCUGACUGAAGACCAGCAGU	3.72
2	hsa-miR-130b	MIMAT0000691	CAGUGCAAUGAUGAAAGGGCAU	59.04
3	**hsa-miR-151-5P**	MIMAT0004697	UCGAGGAGCUCACAGUCUAGU	29.71
4	**hsa-miR-195**	MIMAT0000461	UAGCAGCACAGAAAUAUUGGC	3.52
5	hsa-miR-19a	MIMAT0000073	UGUGCAAAUCUAUGCAAAACUGA	5.53
6	**hsa-miR-20a**	MIMAT0000075	UAAAGUGCUUAUAGUGCAGGUAG	2.31
7	hsa-miR-296	MIMAT0000690	AGGGCCCCCCCUCAAUCCUGU	43.17
8	hsa-miR-29c	MIMAT0000681	UAGCACCAUUUGAAAUCGGUUA	2.8
9	**hsa-miR-30d**	MIMAT0000245	UGUAAACAUCCCCGACUGGAAG	4.56
10	**hsa-miR-328**	MIMAT0000752	CUGGCCCUCUCUGCCCUUCCGU	2.02
11	hsa-miR-339-3p	MIMAT0004702	UGAGCGCCUCGACGACAGAGCCG	14
12	**hsa-miR-362-3p**	MIMAT0004683	AACACACCUAUUCAAGGAUUCA	13.74
13	**hsa-miR-486**	MIMAT0002177	UCCUGUACUGAGCUGCCCCGAG	281.67
14	hsa-miR-505*	MIMAT0004776	GGGAGCCAGGAAGUAUUGAUGU	36.62
15	hsa-miR-579	MIMAT0003244	UUCAUUUGGUAUAAACCGCGAUU	18.64
16	hsa-miR-601	MIMAT0003269	UGGUCUAGGAUUGUUGGAGGAG	4.12
17	hsa-miR-660	MIMAT0003338	UACCCAUUGCAUAUCGGAGUUG	2.84
18	hsa-miR-9*	MIMAT0000442	AUAAAGCUAGAUAACCGAAAGU	4.74
19	**hsa-miR-92a**	MIMAT0000092	UAUUGCACUUGUCCCGGCCUGU	2.57
20	**mmu-miR-451**	MIMAT0001631	AAACCGUUACCAUUACUGAGUU	2.57

Data was normalized using global normalization and was compared with healthy controls and orthopedic injury samples. Data was adjusted for multiple comparisons using adjusted p value  ≤ 0.05 calculated using Benjamin Hochberg algorithm.
