# Supplementary material for: A Panel of Serum MiRNA Biomarkers for the Diagnosis of Severe to Mild Traumatic Brain Injury in Humans
Source: Sci Rep. 2016 Jun 24;6:28148. doi: 10.1038/srep28148 (PMC4919667; doi:10.1038/srep28148)
Supplement: Supplementary Information [file srep28148-s1.doc]

**Supplementary Data**

**Manuscript Title: A Panel of Serum MiRNA Biomarkers for the Diagnosis of Severe to Mild Traumatic Brain Injury in Humans**

**Authors**

Manish Bhomia^1^, Nagaraja S Balakathiresan^1^, Kevin K Wang^2^, Linda Papa^3^ and Radha K Maheshwari^1^.

1. Department of Pathology, Uniformed Services University of the Health Sciences, Bethesda, MD, 20814.
2. Program for Neurotrauma, Neuroproteomics & Biomarker Research, Department of Psychiatry, McKnight Brain Institute, University of Florida, Gainesville, FL 32611
3. Department of Emergency Medicine, Orlando Regional Medical Center, Orlando, Florida, 32806

**Supplementary Table 1**: Total MiRNAs altered in serum samples of MTBI after normalizing with healthy controls. Data was normalized using global normalization and was compared with healthy controls. Data was adjusted for multiple comparisons using adjusted p value <0.05 calculated using Benjamin Hochberg algorithm

| **MTBI vs Control** | | | | | |
| --- | --- | --- | --- | --- | --- |
| S# | Detector | RQ_ mTBI-Control | adj.P.Val_ mTBI-Control | P.Value_ mTBI-Control | GeneSymbol |
| 1 | hsa-miR-381-000571 | 2255.75 | 0.01 | 0.01 | hsa-miR-381 |
| 2 | hsa-miR-185-002271 | 605.52 | 0.00 | 0.00 | hsa-miR-185 |
| 3 | hsa-miR-486-001278 | 523.46 | 0.01 | 0.00 | hsa-miR-486 |
| 4 | hsa-miR-532-001518 | 492.81 | 0.00 | 0.00 | hsa-miR-532 |
| 5 | hsa-miR-423-5p-002340 | 415.56 | 0.00 | 0.00 | hsa-miR-423 |
| 6 | hsa-miR-193a-5p-002281 | 221.14 | 0.00 | 0.00 | hsa-miR-193a |
| 7 | hsa-miR-133a-002246 | 75.25 | 0.02 | 0.01 | hsa-miR-133a |
| 8 | hsa-miR-638-001582 | 46.48 | 0.05 | 0.03 | hsa-miR-638 |
| 9 | hsa-miR-151-5P-002642 | 45.52 | 0.03 | 0.02 | hsa-miR-151 |
| 10 | hsa-miR-223#-002098 | 42.61 | 0.01 | 0.01 | hsa-miR-223 |
| 11 | hsa-miR-625#-002432 | 40.51 | 0.03 | 0.03 | hsa-miR-625 |
| 12 | hsa-miR-505#-002087 | 33.39 | 0.04 | 0.03 | hsa-miR-505 |
| 13 | hsa-miR-194-000493 | 31.43 | 0.04 | 0.03 | hsa-miR-194 |
| 14 | hsa-miR-576-3p-002351 | 25.40 | 0.02 | 0.01 | hsa-miR-576 |
| 15 | hsa-miR-1255B-002801 | 19.19 | 0.01 | 0.00 | hsa-miR-1255B |
| 16 | hsa-miR-362-3p-002117 | 14.54 | 0.01 | 0.01 | hsa-miR-362 |
| 17 | hsa-miR-409-3p-002332 | 12.83 | 0.02 | 0.01 | hsa-miR-409 |
| 18 | mmu-miR-451-001141 | 8.37 | 0.00 | 0.00 | mmu-miR-451 |
| 19 | hsa-miR-16-000391 | 7.44 | 0.00 | 0.00 | hsa-miR-16 |
| 20 | hsa-miR-365-001020 | 6.76 | 0.01 | 0.01 | hsa-miR-365 |
| 21 | hsa-miR-25-000403 | 6.71 | 0.00 | 0.00 | hsa-miR-25 |
| 22 | hsa-miR-151-3p-002254 | 6.61 | 0.02 | 0.01 | hsa-miR-151 |
| 23 | hsa-miR-376c-002122 | 5.21 | 0.00 | 0.00 | hsa-miR-376c |
| 24 | hsa-miR-21-000397 | 4.95 | 0.00 | 0.00 | hsa-miR-21 |
| 25 | hsa-miR-146a-000468 | 4.25 | 0.00 | 0.00 | hsa-miR-146a |
| 26 | hsa-miR-20a-000580 | 4.19 | 0.00 | 0.00 | hsa-miR-20a |
| 27 | hsa-miR-484-001821 | 3.89 | 0.00 | 0.00 | hsa-miR-484 |
| 28 | hsa-miR-92a-000431 | 3.77 | 0.00 | 0.00 | hsa-miR-92a |
| 29 | hsa-miR-152-000475 | 3.64 | 0.00 | 0.00 | hsa-miR-152 |
| 30 | hsa-miR-590-5p-001984 | 3.27 | 0.04 | 0.03 | hsa-miR-590 |
| 31 | hsa-miR-199a-3p-002304 | 3.02 | 0.00 | 0.00 | hsa-miR-199a |
| 32 | hsa-miR-30d-000420 | 2.92 | 0.00 | 0.00 | hsa-miR-30d |
| 33 | hsa-miR-223-002295 | 2.65 | 0.02 | 0.02 | hsa-miR-223 |
| 34 | hsa-miR-186-002285 | 2.57 | 0.00 | 0.00 | hsa-miR-186 |
| 35 | hsa-miR-328-000543 | 2.56 | 0.00 | 0.00 | hsa-miR-328 |
| 36 | hsa-miR-27b-000409 | 2.51 | 0.00 | 0.00 | hsa-miR-27b |
| 37 | hsa-miR-195-000494 | 2.46 | 0.01 | 0.01 | hsa-miR-195 |
| 38 | hsa-miR-27a-000408 | 2.06 | 0.00 | 0.00 | hsa-miR-27a |
| 39 | hsa-miR-19b-000396 | 2.06 | 0.04 | 0.03 | hsa-miR-19b |

**Supplementary Table 2**: Total MiRNAs altered in serum samples of STBI after normalizing with healthy controls. Data was normalized using global normalization and was compared with healthy controls. Data was adjusted for multiple comparisons using adjusted p value <0.05 calculated using Benjamin Hochberg algorithm.

| **sTBI vs Control** | | | | | |
| --- | --- | --- | --- | --- | --- |
| S# | Detector | RQ_ sTBI-Control | adj.P.Val_ sTBI-Control | P.Value_ sTBI-Control | GeneSymbol |
| 1 | hsa-miR-193a-5p-002281 | 476.64 | 0.00 | 0.00 | hsa-miR-193a |
| 2 | hsa-miR-486-001278 | 281.67 | 0.01 | 0.01 | hsa-miR-486 |
| 3 | hsa-miR-423-5p-002340 | 207.23 | 0.01 | 0.00 | hsa-miR-423 |
| 4 | hsa-miR-532-001518 | 202.24 | 0.01 | 0.01 | hsa-miR-532 |
| 5 | hsa-miR-185-002271 | 92.99 | 0.04 | 0.03 | hsa-miR-185 |
| 6 | hsa-miR-133a-002246 | 82.04 | 0.01 | 0.01 | hsa-miR-133a |
| 7 | hsa-miR-576-3p-002351 | 74.38 | 0.00 | 0.00 | hsa-miR-576 |
| 8 | hsa-miR-130b-000456 | 59.04 | 0.04 | 0.03 | hsa-miR-130b |
| 9 | hsa-miR-296-000527 | 43.17 | 0.02 | 0.01 | hsa-miR-296 |
| 10 | hsa-miR-505#-002087 | 36.62 | 0.01 | 0.00 | hsa-miR-505 |
| 11 | hsa-miR-223#-002098 | 34.41 | 0.02 | 0.01 | hsa-miR-223 |
| 12 | hsa-miR-151-5P-002642 | 29.71 | 0.05 | 0.03 | hsa-miR-151 |
| 13 | hsa-miR-579-002398 | 18.64 | 0.01 | 0.01 | hsa-miR-579 |
| 14 | hsa-miR-339-3p-002184 | 14.00 | 0.03 | 0.02 | hsa-miR-339 |
| ***15** | hsa-miR-362-3p-002117 | 13.74 | 0.05 | 0.04 | hsa-miR-362 |
| 16 | hsa-miR-365-001020 | 12.41 | 0.00 | 0.00 | hsa-miR-365 |
| 17 | hsa-miR-29a-002112 | 6.90 | 0.00 | 0.00 | hsa-miR-29a |
| 18 | hsa-miR-19a-000395 | 5.53 | 0.02 | 0.01 | hsa-miR-19a |
| 19 | hsa-miR-9#-002231 | 4.74 | 0.00 | 0.00 | hsa-miR-9 |
| 20 | hsa-miR-30d-000420 | 4.56 | 0.00 | 0.00 | hsa-miR-30d |
| 21 | hsa-miR-25-000403 | 4.22 | 0.00 | 0.00 | hsa-miR-25 |
| 22 | hsa-miR-601-001558 | 4.12 | 0.03 | 0.02 | hsa-miR-601 |
| 23 | hsa-miR-16-000391 | 4.01 | 0.00 | 0.00 | hsa-miR-16 |
| 24 | hsa-miR-1291-002838 | 3.72 | 0.02 | 0.01 | hsa-miR-1291 |
| 25 | hsa-miR-21-000397 | 3.69 | 0.00 | 0.00 | hsa-miR-21 |
| 26 | hsa-miR-195-000494 | 3.52 | 0.00 | 0.00 | hsa-miR-195 |
| 27 | hsa-miR-146a-000468 | 3.12 | 0.01 | 0.00 | hsa-miR-146a |
| 28 | hsa-miR-660-001515 | 2.84 | 0.01 | 0.01 | hsa-miR-660 |
| 29 | hsa-miR-29c-000587 | 2.80 | 0.01 | 0.00 | hsa-miR-29c |
| 30 | hsa-miR-19b-000396 | 2.63 | 0.01 | 0.00 | hsa-miR-19b |
| 31 | mmu-miR-451-001141 | 2.57 | 0.05 | 0.03 | mmu-miR-451 |
| 32 | hsa-miR-92a-000431 | 2.57 | 0.00 | 0.00 | hsa-miR-92a |
| 33 | hsa-miR-186-002285 | 2.56 | 0.02 | 0.02 | hsa-miR-186 |
| 34 | hsa-miR-484-001821 | 2.49 | 0.00 | 0.00 | hsa-miR-484 |
| 35 | hsa-miR-20a-000580 | 2.31 | 0.01 | 0.01 | hsa-miR-20a |
| 36 | hsa-miR-24-000402 | 2.25 | 0.00 | 0.00 | hsa-miR-24 |
| 37 | hsa-miR-328-000543 | 2.02 | 0.02 | 0.01 | hsa-miR-328 |

**Supplementary Table 3**: Total MiRNAs altered in serum samples of Orthopedic Injury group after normalizing with healthy controls. Data was normalized using global normalization and was compared with healthy controls. Data was adjusted for multiple comparisons using adjusted p value <0.05 calculated using Benjamin Hochberg algorithm.

| **Ortho vs Control** | | | | | |
| --- | --- | --- | --- | --- | --- |
| S# | Detector | RQ_ Ortho-Control | adj.P.Val_ Ortho-Control | P.Value_ Ortho-Control | GeneSymbol |
| 1 | hsa-miR-520c-3p-002400 | 23063.46 | 0.02 | 0.00 | hsa-miR-520c |
| 2 | hsa-miR-155-002623 | 941.78 | 0.01 | 0.00 | hsa-miR-155 |
| 3 | hsa-miR-185-002271 | 467.20 | 0.03 | 0.00 | hsa-miR-185 |
| 4 | hsa-miR-766-001986 | 425.82 | 0.01 | 0.00 | hsa-miR-766 |
| 5 | hsa-miR-532-001518 | 366.99 | 0.01 | 0.00 | hsa-miR-532 |
| 6 | hsa-miR-193a-5p-002281 | 322.15 | 0.01 | 0.00 | hsa-miR-193a |
| 7 | hsa-miR-423-5p-002340 | 216.43 | 0.03 | 0.00 | hsa-miR-16 |
| 8 | hsa-miR-132-000457 | 197.50 | 0.03 | 0.01 | hsa-miR-132 |
| 9 | hsa-miR-133a-002246 | 49.67 | 0.03 | 0.02 | hsa-miR-133a |
| 10 | hsa-miR-223#-002098 | 42.32 | 0.03 | 0.01 | hsa-miR-223 |
| 11 | hsa-miR-642-001592 | 27.33 | 0.03 | 0.02 | hsa-miR-642 |
| 12 | hsa-miR-576-3p-002351 | 22.18 | 0.04 | 0.02 | hsa-miR-576 |
| 13 | hsa-miR-409-3p-002332 | 16.73 | 0.04 | 0.03 | hsa-miR-409 |
| 14 | hsa-miR-375-000564 | 16.69 | 0.03 | 0.02 | hsa-miR-375 |
| 15 | hsa-miR-146a-000468 | 12.84 | 0.00 | 0.00 | hsa-miR-146a |
| 16 | hsa-miR-29a-002112 | 10.45 | 0.03 | 0.01 | hsa-miR-29a |
| 17 | hsa-miR-186-002285 | 9.91 | 0.03 | 0.01 | hsa-miR-186 |
| 18 | hsa-miR-376c-002122 | 8.41 | 0.02 | 0.00 | hsa-miR-376c |
| 19 | hsa-miR-197-000497 | 6.62 | 0.00 | 0.00 | hsa-miR-197 |
| 20 | hsa-miR-365-001020 | 6.16 | 0.03 | 0.00 | hsa-miR-365 |
| 21 | hsa-miR-222-002276 | 5.57 | 0.01 | 0.00 | hsa-miR-222 |
| 22 | mmu-miR-374-5p-001319 | 5.29 | 0.03 | 0.00 | mmu-miR-374 |
| 23 | hsa-miR-21-000397 | 4.55 | 0.03 | 0.00 | hsa-miR-21 |
| 24 | hsa-miR-16-000391 | 4.43 | 0.03 | 0.00 | hsa-miR-409 |
| 25 | hsa-miR-192-000491 | 4.30 | 0.03 | 0.01 | hsa-miR-192 |
| 26 | hsa-miR-484-001821 | 4.23 | 0.01 | 0.00 | hsa-miR-484 |
| 27 | hsa-miR-25-000403 | 4.16 | 0.02 | 0.00 | hsa-miR-25 |
| 28 | hsa-miR-223-002295 | 4.05 | 0.03 | 0.01 | hsa-miR-223 |
| 29 | hsa-miR-151-3p-002254 | 3.56 | 0.03 | 0.01 | hsa-miR-151 |
| 30 | hsa-miR-590-5p-001984 | 3.50 | 0.05 | 0.03 | hsa-miR-590 |
| 31 | hsa-miR-24-000402 | 3.48 | 0.01 | 0.00 | hsa-miR-24 |
| 32 | hsa-miR-152-000475 | 2.97 | 0.03 | 0.01 | hsa-miR-152 |
| 33 | hsa-miR-19b-000396 | 2.60 | 0.03 | 0.01 | hsa-miR-19b |
